# Photocatalytic activity of attapulgite–TiO_2_–Ag_3_PO_4_ ternary nanocomposite for degradation of Rhodamine B under simulated solar irradiation

**DOI:** 10.1186/s11671-018-2443-3

**Published:** 2018-01-18

**Authors:** Hongcai He, Zhuolin Jiang, Zhaoling He, Tao Liu, Enzhu Li, Bao-Wen Li

**Affiliations:** 1 State Key Laboratory of Electronic Thin Films and Integrated Devices, University of Electronic Science and Technology of China, Chengdu, 610054 People’s Republic of China; 2 School of Materials Science and Engineering, Wuhan University of Technology, Wuhan, 430070 China

**Keywords:** Photocatalyst, Attapulgite, TiO_2_, Ag_3_PO_4_, Composite

## Abstract

An excellent ternary composite photocatalyst consisting of silver orthophosphate (Ag_3_PO_4_), attapulgite (ATP), and TiO_2_ was synthesized, in which heterojunction was formed between dissimilar semiconductors to promote the separation of photo-generated charges. The ATP/TiO_2_/Ag_3_PO_4_ composite was characterized by SEM, XRD, and UV-vis diffuse reflectance spectroscopy. The co-deposition of Ag_3_PO_4_ and TiO_2_ nanoparticles onto the surface of ATP forms a lath-particle structure. Compared with composite photocatalysts consisting of two phases, ATP/TiO_2_/Ag_3_PO_4_ ternary composite exhibits greatly improved photocatalytic activity for degradation of rhodamine B under simulated solar irradiation. Such ternary composite not only improves the stability of Ag_3_PO_4_, but also lowers the cost by reducing application amount of Ag_3_PO_4_, which provides guidance for the design of Ag_3_PO_4_- and Ag-based composites for photocatalytic applications.

## Background

Organic pollutant degradation has been a critical process towards resolving environmental pollution. Fujishima et al. reported in 1972 that TiO_2_ has the capability of utilizing solar energy for water splitting and hydrogen production [1]. Since then, semiconductor-based photocatalytic technology has become a promising, and yet effective approach to resolve environment pollution. Over the past decades, a number of semiconductors, such as TiO_2_, Ag_3_PO_4_, BiVO_4_, WO_3_, and g-C_3_N_4_, have been extensively investigated for photocatalytic application [2]. Among them, TiO_2_ has received extensive attention due to its good chemical stability, non-photocorrosion, low cost, and nontoxicity. Because of its wide band gap (3.2 eV) and lacking visible light absorption, however, TiO_2_ exhibits low photocatalytic efficiency. The application of TiO_2_-based photocatalysts was thus hampered severely.

The photocatalysts, such as Ag_3_PO_4_ [3], Bi2MoO_6_ [4], WO_3_ [5], and g-C_3_N_4_ [6], can exhibit high-efficiency under visible light irradiation, and thus have drawn extensive research efforts. For example, Ye et al. reported that silver orthophosphate (Ag_3_PO_4_) exhibited much stronger photooxidative capabilities and higher efficiency for photocatalytic degradation [3] than most other known photocatalysts such as WO_3_ [5] and BiVO_4_ [7]. However, the photocatalytic stability of Ag_3_PO_4_ could be deteriorated by the photoreduction of Ag^+^ into metallic Ag. The low photostability and high cost of Ag_3_PO_4_ are concerning issues that will limit its photocatalytic applications. In this context, Ag_3_PO_4_-based composite photocatalysts have been investigated with the goal of improving its photostability and photocatalysis, such as TiO_2_/Ag_3_PO_4_ [8], Ag_3_PO_4_/graphene [9], and Ag_3_PO_4_/Ag/WO_3-*x*_ [10].

Attapulgite (ATP) is a kind of rod-shaped fiber hydrated magnesium aluminum silicate non-metallic mineral, which has remarkable physical and chemical properties, such as exchangeable cations, water absorption, adsorption discoloration, and large specific surface area [11]. ATP is thus considered to be an ideal catalyst carrier with rod morphology, and its high surface area is benefit for absorbing catalyst and pollutant. Although Ag_3_PO_4_- and TiO_2_-based and attapulgite/Ag_3_PO_4_ binary composite photocatalysts have been reported, attapulgite-based ternary composite materials have rarely been investigated.

In this work, the ATP/TiO_2_/Ag_3_PO_4_ ternary composites were synthesized by a facile two-step method for improving the photostability and photocatalysis of Ag_3_PO_4_ and suppressing the consumption of noble metal Ag. The crystalline structure and microstructure of novel ternary composites were characterized by XRD and SEM, respectively, while their photocatalytic activities and stability were measured by degradation of organic dye rhodamine B (RhB) under simulated solar irradiation. This ternary composite exhibits higher photocatalytic efficiency than pure silver phosphate and excellent photocatalytic stability.

## Experimental section

### Materials

**Fig. 1:**
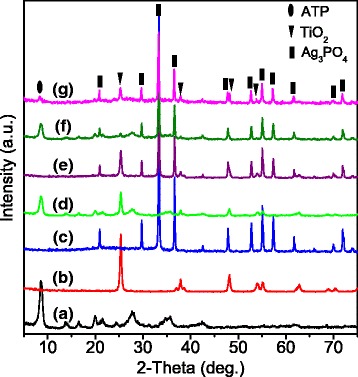
XRD patterns of samples: **a** ATP, **b** TiO_2_, **c** Ag_3_PO_4_, **d** ATP/TiO_2_, **e** Ag_3_PO_4_/TiO_2_, **f** ATP/Ag_3_PO_4_, and **g** ATP/TiO_2_/Ag_3_PO_4_

ATP nanofibers with an average diameter less than 100 nm and an average length less than 1 μm (Fig. 1) was pursued from Jiangsu Qingtao Energy Science and Technology Co., Ltd. RhB (A.R.), EDTA disodium salt dehydrate (GR, 99%), tert-Butanol (GR, ≥ 99.5%), stearyl trimethyl ammonium chloride (STAC, 98%), silver nitrate (AR), and disodium dihydrogen phosphate hydrate (Na_2_HPO_4_·12H_2_O, AR, 99%) were purchased from Macklin. Titanium oxide, anatase (nanopowders, 5–10 nm particle size, 99.8% metals basis, hydrophilic/lipophilic) was purchased from Aladdin.

### Synthesis of samples

The ATP/TiO_2_/Ag_3_PO_4_ ternary composite was synthesized by a facile two-step method. Well-dispersed ATP nanorods and TiO_2_ nanoparticles with mass ratio of 5:2 were first added into deionized water and stirred for 4 h. Through physical and surface electronic absorption, the TiO_2_ nanoparticles were attached to the surface of ATP nanorods. After centrifugal separation, the precipitate was washed with deionized water and then dried at 60 °C for 6 h to obtain ATP/TiO_2_ composites. By a simple precipitation method, Ag_3_PO_4_ nanoparticles were deposited on the surface of ATP/TiO_2_ and ATP/TiO_2_/Ag_3_PO_4_ ternary composites were then prepared. [12] In a typical preparation process, 20 ml silver nitrate solution (0.1 mol/L) was dissolved in ATP/TiO_2_ aqueous suspension with 0.7 g ATP/TiO_2_ composites and 50 ml deionized water by ultrasonic stirring for 30 min. 20 ml Na_2_HPO_4_ aqueous solution (0.1 mol/L) was then added slowly into the above solution with ultrasonic stirring in dark condition for another 40 min. Then, the light yellowish-brown precipitate was centrifuged, washed several times with ethanol absolute, and dried at 60 °C for 12 h, to obtain ATP/TiO_2_/Ag_3_PO_4_ ternary composites. The powder samples of Ag_3_PO_4_, Ag_3_PO_4_/ATP, Ag_3_PO_4_/TiO_2_, and ATP/TiO_2_ were also synthesized using the similar method.

### Characterization

X-ray diffraction was collected using XRD Rigaku D/max-RB) for phase analysis of the powders under 40 kV and 30 mA. The microstructures were evaluated by scanning electron microscopy (SEM, INSPECTF FEI, Netherlands). Ultraviolet-visible (UV-vis) diffuse reflection spectroscopy of the photocatalyst was investigated using U-3010 Hitach UV-vis spectrophotometer using BaSO_4_ as reference.

### Photocatalytic experiment

Photocatalytic degradation of RhB was tested under simulated solar irradiation. 50 mg ATP/TiO_2_/Ag_3_PO_4_ was added to 100 ml RhB solution with a concentration of 5 mg/L and stirred in dark for 40 min to ensure adsorption-desorption equilibrium. The light source was a 300 W Xe lamp (Microsolar300, PerfectLight, Beijing, China) at about 150 mW/cm^2^ (as tested by a radiometer FZ-A, Photoelectric Instrument Factory of Beijing Normal University, China). After opening the lamp, 4 ml solution was taken out at known time intervals and separated through centrifugation (10,000 rpm, 10 min). The supernatants were analyzed by recording variations of absorption peak (554 nm) in the UV-vis spectra using UV/vis spectro-photometer (T6, PERSEE, Beijing, China).

The degradation degree of RhB dye was determined according to the following equation: *D*% = (*c*_*0*_ *− c*)/*c*_*0*_ *×* 100% = (*A*_0_ *− A*)*/A*_0_ *×* 100%, where *c*_0_ and *c* are the initial concentration and concentration after photocatalysis of the solution, respectively; and *A*_0_ and *A* are the absorbance values of the solution before and after photocatalytic reaction, respectively.

## Results and discussion

### Characterization of the ATP-Ag_3_PO_4_-TiO_2_ composites

The XRD patterns of ATP, TiO_2_, Ag_3_PO_4_, and nanocomposites are shown in Fig. 1. The diffraction peaks in Fig. 1a can be indexed as ATP phase with monoclinic structure (JCPDS # 21–0958), which implies that the ATP had been specially purified and no impurity phases exist. Figure 1b displays typical diffraction peaks of anatase TiO_2_ without any impurities, while Fig. 1c shows the diffraction peaks corresponding to pure Ag_3_PO_4_ phase, in good agreement with JCPDS # 06-0505. There are not any impurity phases or structure destabilization for all the nanocomposite samples of ATP/TiO_2_ (Fig. 1d), Ag_3_PO_4_/TiO_2_ (Fig. 1e), ATP/Ag_3_PO_4_ (Fig. 1f), and ATP/TiO_2_/Ag_3_PO_4_ (Fig. 1g). In XRD patterns of ATP/TiO_2_/Ag_3_PO_4_ (Fig. 1g), main characteristic peaks associated with both Ag_3_PO_4_ and TiO_2_ can be detected, while the diffraction peaks from ATP phase are much weaker. The phenomenon implies that the ATP nanorods are cladded by TiO_2_ and Ag_3_PO_4_ nanoparticles.

**Fig. 2:**
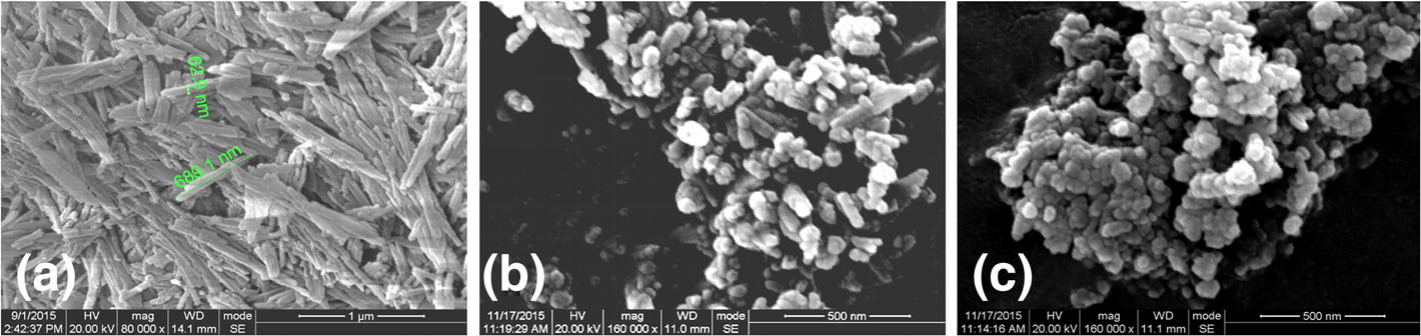
SEM images of **a** ATP, **b** ATP/TiO_2_, and **c** ATP/TiO_2_/Ag_3_PO_4_ powders

The morphological and microstructure of the composite photocatalysts are shown in Fig. 2. ATP nanorods exhibited an average length less than 1 μm and a diameter less than 100 nm (Fig. 2a). Due to surface physical and chemical adsorption, TiO_2_ nanoparticles with diameter of about 40 nm attached to the surface of ATP nanorods and formed ATP/TiO_2_ composites, as shown in Fig. 2b. In Fig. 2c, the ATP nanorods were fully covered by Ag_3_PO_4_ and TiO_2_ particles in ATP/TiO_2_/Ag_3_PO_4_ ternary composite, while Ag_3_PO_4_ appeared on the surface of ATP/TiO_2_ composites in the form of uniform spheroidal particles with diameter of about 50 nm.

### Absorption spectra

**Fig. 3:**
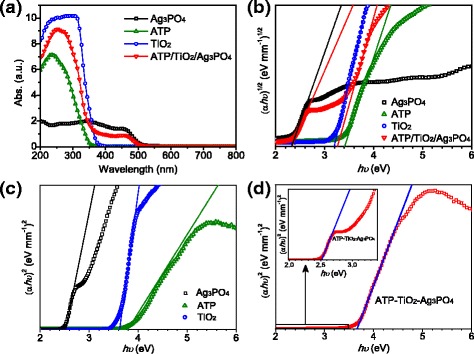
**a** UV-vis absorption spectra and **b** plots of (*αhν*)^1/2^ versus (*hν*) of Ag_3_PO_4_, ATP, TiO_2_ and ATP/TiO_2_/Ag_3_PO_4_ ternary nanocomposite; **c** plots of (*αhν*)^2^ versus (*hν*) of Ag_3_PO_4_, ATP and TiO_2_; **d** plots of (*αhν*)^2^ versus (*hν*) of ATP/TiO_2_/Ag_3_PO_4_ ternary nanocomposite, and the inset in **d** is the partial enlarged detail of the plots in **d**

The UV-vis absorption spectra of Ag_3_PO_4_, ATP, TiO_2_, and ATP/TiO_2_/Ag_3_PO_4_ are shown in Fig. 3a. Similar with the reported results, [3] Ag_3_PO_4_ exhibits good absorption from the UV to the visible light region with a wavelength up to about 500 nm. On the other hand, TiO_2_ exhibits an excellent UV absorption without obvious absorption in visible light region. ATP shows a lower UV absorption and little absorption in visible light region. As expected, ATP/TiO_2_/Ag_3_PO_4_ ternary nanocomposite exhibits a strong UV absorption benefiting from TiO_2_ and ATP and the enhanced visible-light absorption imposed by Ag_3_PO_4_. The optical band gap (*E*_g_) can be estimated from the optical absorption edge according to the Eq. (1). [13, 14]

1 $$ \alpha hv=A{\left( hv-{E}_g\right)}^m, $$

where *α* is the spectral absorption coefficient, “*hv*” is the photon energy, *A* is a constant, and *m* is equal to 0.5 or 2 for direct and indirect transitions, respectively. TiO_2_ [15] is generally regarded as an indirect bandgap semiconductor, and its indirect *E*_g_ is determined by the interception of a straight line fitted through the low-energy side of the curve (*αhυ*)^1/2^ versus *hυ* as shown in Fig. 3b, with an estimated value of about 3.20 eV. Ag_3_PO_4_ was reported as an indirect bandgap semiconductor, and its direct gap at the Gamma point and the indirect gap are very close in terms of the calculated results. [16] Its direct gap of about 2.45 eV was regarded as the bandgap of Ag_3_PO_4_ in most reports. Here, the indirect *E*_g_ and direct *E*_g_ are determined by the interception of the straight line fitted through the low-energy side of the curve (*αhυ*)^1/m^ (m = 2 and 0.5) versus *hυ*, respectively. The results of Ag_3_PO_4_ reveal an indirect bandgap of 2.33 eV (Fig. 3b) and a direct bandgap of 2.49 eV (Fig. 3c). The direct *E*_g_ of 2.49 eV is more matched with its absorption band edge than the indirect bandgap of 2.33 eV. Thus, the *E*_g_ of Ag_3_PO_4_ is determined as 2.49 eV. Similarly, ATP shows an indirect bandgap of 3.37 eV (Fig. 3b) and a direct bandgap of 3.75 eV (Fig. 3c), and the *E*_g_ of ATP is determined as 3.75 eV. The above bandgap values of TiO_2_, Ag_3_PO_4_ and ATP are quite close to the reported results. [17] In the ATP/TiO_2_/Ag_3_PO_4_ ternary nanocomposite, there are two different optical absorption band edges of about 385 and 510 nm in the UV-vis absorption spectra, from which two different *E*_g_ values can be estimated. From the absorption band edge of 385 nm, a direct *E*_g_ of about 3.64 eV is obtained, which is in between those of TiO_2_ and ATP as a result of composite effect. Corresponding to the absorption edge of 510 nm, a direct *E*_g_ of about 2.49 eV is obtained, in according with the direct *E*_g_ of Ag_3_PO_4_. As a result, the ternary composite retained the similar outstanding absorption in visible light as Ag_3_PO_4_, as well as good UV absorption derived from TiO_2_ and ATP. This result implies the ATP/TiO_2_/Ag_3_PO_4_ ternary composite has the potential to be an excellent photocatalyst in the wavelength range from UV light to visible light.

### Photocatalytic activities

**Fig. 4:**
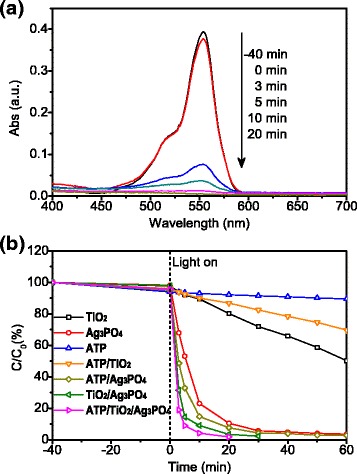
**a** UV-vis absorption spectra of the photocatalytic degraded RhB solutions by the ATP/TiO_2_/Ag_3_PO_4_ ternary nanocomposite at different times. **b** Photocatalytic degradation of RhB with different photocatalysts under simulated solar irradiation

The photocatalytic activity of the resulting samples was evaluated by the degradation of RhB under Xe light irradiation, Fig. 4. After immersing photocatalysts, RhB solutions were stirred for 40 min in dark condition to establish adsorption-desorption equilibrium with the goal of eliminating the interference of adsorption. Figure 4a shows the evolution of absorption spectra during the photodegradation of RhB solutions by ATP/TiO_2_/Ag_3_PO_4_ ternary nanocomposite under Xe light illumination as a function of time. The absorption peaks centered at 554 nm correspond to the characteristic absorption peak of RhB. Due to the photodegradation of RhB, the peak strength decreased as the concentration of RhB decreased. After stirring the solution for 40 min in dark condition, only a little decrease in the absorption peak intensity is observed for RhB, which indicates a weak dye adsorption of the nanocomposite. After irradiation for 20 min, the characteristic absorption peak of RhB nearly disappeared, implying almost complete degradation of the dye in the solution. Under similar Xe light irradiation condition, the photocatalytic degradation of RhB with different photocatalysts is compared in Fig. 4b. The photocatalysts of single-phase TiO_2_ and ATP showed lower degradation rate than 50% under 60 min irradiation, while Ag_3_PO_4_ displayed much stronger and faster photocatalytic degradation, in good agreement with previous reports on photocatalysis of TiO_2_ and Ag_3_PO_4_ [18]. Ag_3_PO_4_ was reported as a strong photocatalyst, but its stability of photocatalytic activity is low and its cost is high. The ternary nanocomposites revealed a fast degradation rate of around 81.1% only after 3 min irradiation and almost complete degradation after 20 min irradiation, which are obviously higher than that of single-phase Ag_3_PO_4_ and other binary composite photocatalysts including ATP/Ag_3_PO_4_ and TiO_2_/Ag_3_PO_4_ as seen in Fig. 4b. ATP has little photocatalytic activity, but it has been reported with good ability of adsorption, [19] which facilitates dye molecules adhering to its surface, and results in a higher degradation rate of RhB by the ATP/TiO_2_/Ag_3_PO_4_ ternary nanocomposite photocatalysts. Interestingly, the ATP/TiO_2_/Ag_3_PO_4_ photocatalysts showed stronger photocatalytic degradation efficiency than TiO_2_/Ag_3_PO_4_ or Ag_3_PO_4_ with the same weight. As a result, the application amount of high-cost Ag_3_PO_4_ is reduced.

**Fig. 5:**
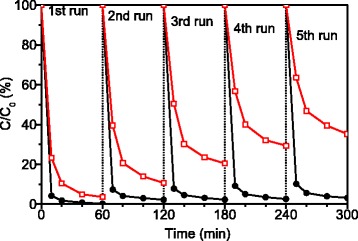
Repeated photocatalytic degradation of RhB with Ag_3_PO_4_ (red open squares) and ATP/TiO_2_/Ag_3_PO_4_ ternary composites (black solid circles) under simulated solar irradiation

The stability of the photocatalysts for photodegradation of RhB under Xe light irradiation was evaluated by repeated photocatalytic experiments. Similar test was also performed on Ag_3_PO_4_ for comparison. After each run of photocatalytic degradation, the photocatalysts were separated, washed, dried, and then recycled for the next run. The initial concentration of RhB and the dosage of photocatalyst were kept consistent during each run of photocatalytic degradation. The results are shown in Fig. 5. After every run, the activity of Ag_3_PO_4_ significantly decreased as expected [20]. In the photocatalytic process, the active sites were covered by Ag appearing on the surface of Ag_3_PO_4_ particles. The photocatalytic activity of the ATP/TiO_2_/Ag_3_PO_4_ ternary nanocomposite remained unchanged even after five cycling runs of photodegradation of RhB. This result indicates that the photocatalysis is very stable in ATP/TiO_2_/Ag_3_PO_4_ ternary nanocomposites.

### Possible mechanism in photocatalytic process

**Fig. 6:**
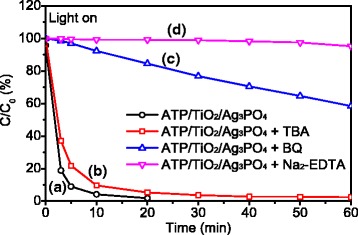
Reactive species trapping experiments of ATP/TiO_2_/Ag_3_PO_4_ composite photocatalyst

In photocatalytic degradation processes, the common reactive oxygen species include •OH radicals, O_2_^•–^ radicals and holes (h^+^). [2] The trapping experiments were carried out to monitor the reactive oxygen species involved in photocatalytic process of ATP/TiO_2_/Ag_3_PO_4_ composites over RhB. Three chemicals of tert-butanol (TBA), benzoquinone (BQ), and disodium ethylenediaminetetraacetate (Na_2_-EDTA) were used as scavengers of •OH radicals, O_2_^•–^ radicals and holes, respectively. [9] The experimental results under Xe light irradiation are shown in Fig. 6. The introduction of 1 mM TBA (•OH radical scavenger) has no obvious influence on the photocatalytic activity of the composite photocatalyst (Fig. 6b). This result indicated that OH· radicals are not the main active oxygen species in the photocatalytic process. The addition of 1 mM BQ (O_2_^•–^ radical scavenger) reduces the photocatalytic degradation degree of RhB to 42% in 60 min (Fig. 6c), which indicates that O_2_^•–^ radicals make an important but only segmental contribution to photocatalytic performance. After adding the hole scavenger Na_2_-EDTA (1 mM) into the photocatalytic system, the photocatalytic degradation activity of ATP/TiO_2_/Ag_3_PO_4_ nanocomposites is almost completely suppressed (Fig. 6d), and the degradation degree of RhB decreases to less than 5% after 60 min. This result implies that holes play a key role in photocatalytic degradation. In consequence, holes and O_2_^•–^ radicals are the main reactive radicals in the ATP/TiO_2_/Ag_3_PO_4_ photocatalytic process degrading RhB under Xe light irradiation.

**Fig. 7:**
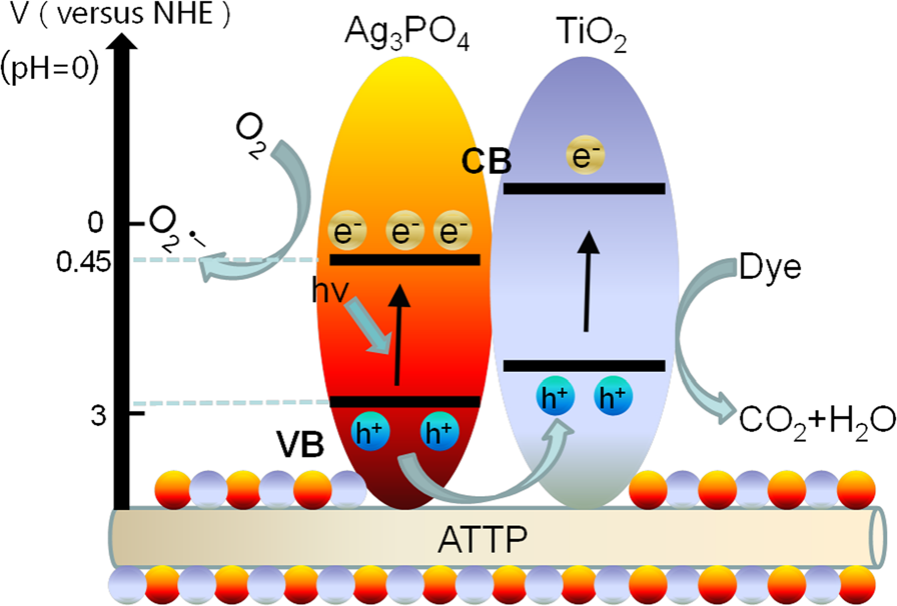
Proposed photocatalytic mechanism of ATP/TiO_2_/Ag_3_PO_4_ composites

Based on the discussion mentioned above, a possible photocatalytic mechanism was proposed to explain the photocatalytic degradation of RhB by ATP/TiO_2_/Ag_3_PO_4_ ternary composite photocatalysts, as shown in Fig. 7. The potentials for conduction band (CB) and valence band (VB) of TiO_2_ are − 0.5 eV vs. NHE, and + 2.70 eV vs. NHE, respectively [21, 22]. These values are more negative than that of both Ag_3_PO_4_ (CB + 0.45 eV vs. NHE, VB + 2.97 eV vs. NHE) [3, 16] and ATP (CB − 0.25 eV vs. NHE, VB + 3.50 eV vs. NHE). Therefore, the photo-generated electrons in the CB of TiO_2_ can easily transfer to that of Ag_3_PO_4_, while the photo-induced holes in the VB of Ag_3_PO_4_ will migrate to that of TiO_2_, which promotes the effective separation of photo-generated electron–hole pairs and decreases the recombination probability of electrons and holes. As a result, the ATP/TiO_2_/Ag_3_PO_4_ composite photocatalyst can exhibit higher photocatalytic activities than single phase Ag_3_PO_4_. Meanwhile, the holes in VB of TiO_2_, which has strong oxidation characteristics, not only could significantly accelerate the photocatalytic reaction rates of RhB degradation, but also could oxidize H_2_O to generate O_2_. The reduction potential of O_2_^•–^ is − 0.28 eV, while the potentials of CB for TiO_2_ and Ag_3_PO_4_ are − 0.3 and + 0.45 eV, respectively. Therefore, the resulting O_2_ at the surface of photocatalysts then could capture photogenerated electrons to produce O_2_^•–^ radicals, and the Ag^+^ ions in Ag_3_PO_4_ could be protected from photoreduction into metallic Ag (Ag^+^ + e^−^ → Ag) since the electrons were consumed in the reaction with O_2_. In consequence, the composite photocatalyst with TiO_2_ and Ag_3_PO_4_ shows much higher stability than single-phase Ag_3_PO_4_ photocatalyst.

## Conclusions

In conclusion, we synthesized ATP/TiO_2_/Ag_3_PO_4_ ternary composite through a simple method: TiO_2_ nanoparticles were absorbed on the surface of ATP to form a binary structure, and then Ag_3_PO_4_ nanoparticles were deposited on ATP/TiO_2_ composite through electrostatic interaction. The heterogeneous junction formed in the ternary composite improves the photocatalytic efficiency and stability. In comparison with pure Ag_3_PO_4_ phase, this kind of composite photocatalyst not only reduces the consumption of the precious metal silver to a larger extent, but also improves the efficiency of photocatalysts. Our results will provide guidance to design Ag-based composites for photocatalytic application.

## Abbreviations

ATP: Attapulgite
BQ: Benzoquinone
CB: Conduction band
Na_2_-EDTA: Disodium ethylenediaminetetraacetate
RhB: Rhodamine B
TBA: Tert-butanol
VB: Valence band
